# 

**Published:** 1877-01

**Authors:** 


### CONTENTS.

### COMMUNICATIONS

| | |
|---|---|
| Treatment of Teeth During Gestation and Lactation . . . . . . | 9 |
| Address . . . . . . . | 15 |
| Dental College Education . . . . | 21 |
| Carbolic Acid . . . . . . | 26 |
| Hypodermic Injections of Carbolic Acid in Neuralgia . . . . . | 27 |
| Trans- and Replantation of Teeth . . | 29 |
| Camphor in Ill-Conditioned Wounds . | 30 |
| The Principles of Antiseptic Surgery . | 31 |
| Finishing Fillings . . . . . | 40 |
| Treatment of a Suppurating Tooth Socket . | 45 |
| Minute Subdivision of Coloring Matter . | 48 |
| Hygroscopic Paper . . . . . . | 48 |

### EDITORIAL.

| | |
|---|---|
| Thirty-First Volume . . . . . | 49 |
| Ohio State Dental Society . . . | 50 |
| Continuous Gum Dentures . . . | 51 |

